# Supplementary material for: EnzML: multi-label prediction of enzyme classes using InterPro signatures
Source: BMC Bioinformatics. 2012 Apr 25;13:61. doi: 10.1186/1471-2105-13-61 (PMC3483700; doi:10.1186/1471-2105-13-61)
Supplement: Addtional file 5 — The Java code to format the data files, evaluate and predict. The file enzml_java_code.tar.gz contains the Java code used to format database data to ARFF and XML formats, to execute cross and train-test (jackknife) evaluations and to record evaluation results to database. More information is included in the readme.txt file and the Javadoc files. The code can be used with a MySQL database. To use a different database software, other JDBC drivers might be required. [file 1471-2105-13-61-S5.gz › java_code/enzml2011/doc/uk/ac/ed/inf/enzml/weka/ArffPropsTableManager.html]

ArffPropsTableManager


---


|  |  |  |
| --- | --- | --- |
| **Overview** \| **Package** \| **Class** \| **Use** \| **Tree** \| **Deprecated** \| **Index** \| **Help** | |  |
| **PREV CLASS**   **NEXT CLASS** | **FRAMES**    **NO FRAMES**     **All Classes** |  |
| SUMMARY: NESTED \| FIELD \| CONSTR \| METHOD | DETAIL: FIELD \| CONSTR \| METHOD |  |


---


## uk.ac.ed.inf.enzml.weka Class ArffPropsTableManager

```
java.lang.Object
  uk.ac.ed.inf.utils.database.DbManaged
      uk.ac.ed.inf.utils.database.TableManager
          uk.ac.ed.inf.enzml.weka.ArffPropsTableManager
```

---

``` public class ArffPropsTableManager extends uk.ac.ed.inf.utils.database.TableManager ```

Writes into a database table the pointers needed to build an arff file (the
name of the arff file, the query to select instances, attributes, class
values etc)
TODO integrate arff recording to database with mulan experiment recording

**Version:**
:   16 Jun 2010

**Author:**
:   Luna De Ferrari luna.deferrari-at-ed.ac.uk

---

**Field Summary**

**Fields inherited from class uk.ac.ed.inf.utils.database.TableManager**

`m_writer`


**Constructor Summary**

`ArffPropsTableManager(Arff arffGenerator)`
  
          Constructor 1: initialises the table manager to point to the arff table.

`ArffPropsTableManager(uk.ac.ed.inf.utils.database.DbManager db)`
  
          Constructor 2: initialises the table manager to point to the arff table.


| **Method Summary** | |
| --- | --- |
| `uk.ac.ed.inf.utils.database.TableRow` | `createRow()` |
| `Arff` | `getArffGenerator()` |
| `int` | `getArffRecordId()` |
| `ArffPropsTableReader` | `getReader()` |
| `static void` | `main(java.lang.String[] args)`             Main for recreating table |
| `int` | `writeArffRecordToDb()` |

**Methods inherited from class uk.ac.ed.inf.utils.database.TableManager**

`getTableCreator, getTableName, getTableReader, getTableWriter`

**Methods inherited from class uk.ac.ed.inf.utils.database.DbManaged**

`getDbConnection, getDbManager`

**Methods inherited from class java.lang.Object**

`equals, getClass, hashCode, notify, notifyAll, toString, wait, wait, wait`

**Constructor Detail**

### ArffPropsTableManager

```
public ArffPropsTableManager(Arff arffGenerator)
```

:   Constructor 1: initialises the table manager to point to the arff table.
    Can be used for writing and reading.

---


### ArffPropsTableManager

```
public ArffPropsTableManager(uk.ac.ed.inf.utils.database.DbManager db)
```

:   Constructor 2: initialises the table manager to point to the arff table.
    Can be used for reading only.


**Method Detail**

### createRow

```
public uk.ac.ed.inf.utils.database.TableRow createRow()
```

---


### getArffGenerator

```
public Arff getArffGenerator()
```

---


### getArffRecordId

```
public int getArffRecordId()
```

---


### getReader

```
public ArffPropsTableReader getReader()
```

---


### writeArffRecordToDb

```
public int writeArffRecordToDb()
```

---


### main

```
public static void main(java.lang.String[] args)
```

:   Main for recreating table

    :   **Parameters:**: `args` - **Throws:**: `java.sql.SQLException`


---


|  |  |  |
| --- | --- | --- |
| **Overview** \| **Package** \| **Class** \| **Use** \| **Tree** \| **Deprecated** \| **Index** \| **Help** | |  |
| **PREV CLASS**   **NEXT CLASS** | **FRAMES**    **NO FRAMES**     **All Classes** |  |
| SUMMARY: NESTED \| FIELD \| CONSTR \| METHOD | DETAIL: FIELD \| CONSTR \| METHOD |  |


---
